# Predictors of intact and C-terminal fibroblast growth factor 23 in Gambian children

**DOI:** 10.1530/EC-13-0070

**Published:** 2013-12-19

**Authors:** Vickie Braithwaite, Kerry S Jones, Shima Assar, Inez Schoenmakers, Ann Prentice

**Affiliations:** 1 Medical Research Council (MRC) Human Nutrition ResearchElsie Widdowson LaboratoriesFulbourn Road, Cambridge, CB1 9NLUK; 2 MRC Keneba, KenebaWest KiangThe Gambia

**Keywords:** FGF23, vitamin D half-life, rickets, iron status, children

## Abstract

Elevated C-terminal fibroblast growth factor 23 (C-FGF23) concentrations have been reported in Gambian children with and without putative Ca-deficiency rickets. The aims of this study were to investigate whether i) elevated C-FGF23 concentrations in Gambian children persist long term; ii) they are associated with higher intact FGF23 concentrations (I-FGF23), poor iron status and shorter 25-hydroxyvitamin D half-life (25OHD-*t*_1/2_); and iii) the persistence and predictors of elevated FGF23 concentrations differ between children with and without a history of rickets. Children (8–16 years, *n*=64) with a history of rickets and a C-FGF23 concentration >125 RU/ml (bone deformity (BD), *n*=20) and local community children with a previously measured elevated C-FGF23 concentration (LC+, *n*=20) or a previously measured C-FGF23 concentration within the normal range (LC−, *n*=24) participated. BD children had no remaining signs of bone deformities. C-FGF23 concentration had normalised in BD children, but remained elevated in LC+ children. All the children had I-FGF23 concentration within the normal range, but I-FGF23 concentration was higher and iron status poorer in LC+ children. 1,25-dihydroxyvitamin D was the strongest negative predictor of I-FGF23 concentration (*R*^2^=18%; *P*=0.0006) and soluble transferrin receptor was the strongest positive predictor of C-FGF23 concentration (*R*^2^=33%; *P*≤0.0001). C-FGF23 and I-FGF23 concentrations were poorly correlated with each other (*R*^2^=5.3%; *P*=0.07). 25OHD-*t*_1/2_ was shorter in BD children than in LC− children (mean (s.d.): 24.5 (6.1) and 31.5 (11.5) days respectively; *P*=0.05). This study demonstrated that elevated C-FGF23 concentrations normalised over time in Gambian children with a history of rickets but not in local children, suggesting a different aetiology; that children with resolved rickets had a shorter 25OHD-*t*_1/2_, suggesting a long-standing increased expenditure of 25OHD, and that iron deficiency is a predictor of elevated C-FGF23 concentrations in both groups of Gambian children.

## Introduction

Elevated plasma C-terminal fibroblast growth factor 23 (C-FGF23) concentrations have been recorded in Gambian children with putative Ca-deficiency rickets (1) and, at a lower prevalence, in apparently healthy children from the local community (2). FGF23 is a bone-derived phosphate (P)-regulating hormone that acts primarily in the proximal tubule cells of the kidney. A raised FGF23 concentration is a predictor of mortality in patients with renal failure (3). FGF23 together with its co-receptor α-Klotho (4) binds to the FGF receptor (FGFR) to initiate the internalisation of sodium P transporters NaPi-2a (SLC34A1) and NaPi-2c (SLC34A3) (5). In diseased states where FGF23 concentration is elevated, the net result is an increased urinary P excretion through a decreased reabsorption of P from the glomerular filtrate.

As well as having direct effects on P metabolism, FGF23 targets enzymes involved in vitamin D metabolism. These include cytochrome P450 enzymes CYP27B1 and CYP24A1. CYP27B1 is expressed in the kidney and hydroxylates 25-hydroxyvitamin D (25OHD) to the active vitamin D metabolite 1,25-dihydroxyvitamin D (1,25(OH)_2_D). CYP24A1 is expressed in a variety of tissues including the liver and the kidney and hydroxylates 25OHD to 24,25-dihydroxyvitamin D (24,25(OH)_2_D) and 1,25(OH)_2_D to 1,24,25-trihydroxyvitamin D (1,24,25(OH)_3_D) and to further downstream metabolites. FGF23 down-regulates the activity of CYP27B1 and increases the activity of CYP24A1 (6, 7). Conversely, 1,25(OH)_2_D is a known stimulator of FGF23 production, and two human studies have shown that vitamin D administration can modulate circulating FGF23 concentration (8, 9).

Circulating FGF23 concentration is commonly measured using one of two commercially available assays: the Kainos intact FGF23 (I-FGF23) assay and the Immunotopics C-FGF23 assay. The I-FGF23 assay detects the full-length protein, whereas the C-FGF23 assay binds to epitopes within the C-terminal region of the FGF23 protein and therefore detects both the full-length protein and C-terminal fragments. The C-terminal end of the I-FGF23 protein contains the motif required for binding to the *de novo* α-Klotho–FGFR-binding site, which subsequently allows for the remaining N-terminus of the FGF23 protein to activate the FGFR and to initiate a signal (10). Therefore, the full-length I-FGF23 protein is considered to be the biologically active form of the FGF23 hormone (11). The C-terminal fragment is generally regarded as inactive, although there is some evidence to suggest that C-terminal fragments may have anti-phosphaturic effects in mice (10) or conversely phosphaturic activity in rats (12). In addition, there is a growing body of evidence showing associations between C-FGF23 concentration and iron status (2, 13, 14, 15, 16, 17).

As well as being regulated by FGF23, 1,25(OH)_2_D production is controlled by parathyroid hormone (PTH) (18). PTH is secreted by the parathyroid glands in response to low circulating ionised Ca concentration and PTH promotes CYP27B1 activity. Dietary Ca intake is known to be ubiquitously low in Gambia, and factors that may reduce intestinal Ca absorption such as *Helicobacter pylori* infection (19) and intestinal malabsorption are prevalent in Gambian infants (20). It has been shown that states of Ca deficiency can increase the demand for 25OHD (21). It is, therefore, plausible that circulating FGF23 concentration and dietary intake and absorption of Ca may alter the rate at which 25OHD is utilised.

The aims of this study were to determine longitudinal changes in C-FGF23 concentration and cross-sectional predictors of I-FGF23 and C-FGF23 concentrations in Gambian children with and without a history of rickets and to determine whether circulating FGF23 concentration and dietary Ca intake are determinants of the half-life of 25-hydroxyvitamin D (25OHD-*t*_1/2_).

## Subjects and methods

### Study approvals and consent

Written informed consent was obtained from the parents of the children involved in the study. Ethical approval was given by the Gambian Government/MRC Laboratories Joint Ethics Committee.

### Subjects and study design

All children had participated in a previous study conducted at MRC Keneba 3.2–5.8 years earlier (2, 13). Children aged between 8.0 and 16.0 years were recruited on the basis of a previously determined plasma C-FGF23 concentration with or without a history of rickets-like bone deformities and were allocated to one of three groups. A concentration of C-FGF23 >125 RU/ml (C-FGF23: Immutopics, Inc., San Clemente, CA, USA) was considered elevated. The original diagnosis of rickets was made on the basis of physical signs consistent with rickets and radiographic evidence of rickets and/or an elevated concentration of alkaline phosphatase (1). The children were treated with Ca and vitamin D, which was subsequently terminated after 12 months (1). The bone deformity (BD) group consisted of children with a history of rickets-like bone deformities and a previously measured C-FGF23 concentration >125 RU/ml. The LC+ group consisted of local community (LC) children with no history of rickets-like bone deformities but had a previously measured C-FGF23 concentration >125 RU/ml. Finally, the LC− group consisted of LC children with no history of rickets-like bone deformities and a previously measured C-FGF23 concentration within the normal range (25–125 RU/ml). Children in the LC groups were also selected to age- and sex-match the children in the BD group. None of the children were on current medication or were receiving supplements.

### Anthropometry

Weight was measured to the nearest 0.1 kg using a calibrated electronic scale (HD-305 Tanita, Tanita Europe BV, Naarden, The Netherlands). Standing height and sitting height were measured to the nearest 0.1 cm using a portable stadiometer (Leicester Height Measure, SECA, Hamburg, Germany). Whole-body fat and trunk fat were measured by impedance using a Tanita scale (BC-418MA Tanita, Tanita Europe BV) and expressed as a percentage of total body or trunk mass.

### Fasting blood and urine collection

An overnight-fasted 2-h urine sample and with a mid-point lithium-heparin (LiHep) and EDTA blood sample and a non-fasting 24-h urine sample were obtained as described previously (13). Blood ionised Ca concentration adjusted to a pH of 7.4 (iCa) and derived Hb concentration were measured in whole LiHep-blood samples (ABL77, Radiometer Ltd, Crawley, West Sussex, UK) within 10 min of collection. Plasma was separated from whole blood by centrifugation at 4 °C (20 min at 1800 ***g***) within 30 min and subsequently frozen at −70 °C. The EDTA pellet was used to determine zinc protoporphyrin (ZnPP) concentration on a haematofluorometer (ZPP 206d, Aviv Biomedical, Inc., Lakewood, NJ, USA). Acidified (HCl, 10 μl/ml, laboratory reagent grade SD 1.18, Fisher Scientific, Gillingham, Kent, UK) urine aliquots were stored at −20 °C. All the frozen samples were sent on dry ice to MRC Human Nutrition Research (HNR), Cambridge, UK, and plasma samples were stored at −70 °C and urine samples at −20 °C until analysis.

### Biochemical analysis

The plasma samples were analysed for markers of vitamin D, Ca and P metabolism, iron status and inflammation, and renal and liver function, using commercially available methods according to the manufacturers' instructions. EDTA-plasma samples were used to determine FGF23 concentration by ELISA (C-FGF23: Immutopics, Inc., and I-FGF23 (22): Kainos, Tokyo, Japan) and intact PTH concentration by IRMA (Immulite, Siemens Healthcare Diagnostics, Camberley, Surrey, UK). Concentrations of C-FGF23 >125 RU/ml or I-FGF23 >52 pg/ml were considered elevated (23). LiHep-plasma samples were used for the other assays. These included determination of 25OHD (Diasorin, Stillwater, MN, USA) and 1,25(OH)_2_D (IDS, Boldon, Tyne & Wear, UK) concentrations by RIA and soluble transferrin receptor (sTfR; Ramco Laboratories, Inc., Stafford, TX, USA) concentration by ELISA. A concentration of 25OHD of <25 nmol/l was taken as an indicator of an increased risk of vitamin D-deficiency rickets (24, 25). Total Ca, P, Mg, Cr, albumin (alb), total alkaline phosphatase (TALP) and cystatin C (cys C) concentrations were measured using commercially available colorimetric methods (Dimension Clinical Chemistry Systems, Siemens Healthcare Diagnostics, Camberley, Surrey, UK), C-reactive protein (CRP) concentration by particle-enhanced turbidimetric immunoassay, and α-1-acid glycoprotein (AGP) and ferritin (Ferr) concentrations by ELISA (Dimension Clinical Chemistry Systems, Siemens Healthcare Diagnostics). Ca, P and Cr concentrations were determined in the acidified 2- and 24-h urine samples using colorimetric methods as used for the plasma samples. Urinary Ca and P concentrations are expressed as molar ratios to Cr concentration.

Assay accuracy and precision were monitored across the working range of the assays using reference materials provided by the manufacturer or by external quality assurance schemes. Quality assurance for PTH was monitored by the National External Quality Assessment Service (Department of Clinical Biochemistry, Royal Infirmary, Edinburgh, UK) and for 25OHD and 1,25(OH)_2_D by the Vitamin D External Quality Assessment Scheme (Endocrine/Oncology Laboratory, Charring Cross Hospital, London, UK). For sTfR, the internal standard for quality assessment (QA) was an in-house human plasma sample and the external standard for QA was obtained from the Centre for Disease Controls (CDC) USA. For all other analyses, in-house standards were used for QA.

### Dietary assessment, infection status and intestinal integrity

Two-day weighed dietary assessment was conducted using Gambian food composition tables (26) in the same way as described previously (13).

*H. pylori* infection was determined using a non-invasive stable isotope urea breath test. Fifty micrograms of ^13^C-urea (Cambridge Isotopes, Tewsbury, MA, USA) in a 100 ml solution of polycal (10% w/v, Nutricia, Trowbridge, Wiltshire, UK) were given to each subject. Two baseline breath samples were collected into gas tubes (12 ml, Labco Ltd., Ceredigion, UK) from overnight-fasted subjects, after which the subjects were provided with the urea dose to drink. After 30 min, two more breath samples were collected. The gas tubes were kept at room temperature and were analysed at MRC HNR using isotope ratio mass spectrometry (IRMS) (AP2003 IRMS, Analytical Precision Products, Ltd., Cambridge, UK). A delta over baseline (DOB) >5.47‰ was considered to be indicative of the presence of *H. pylori* (27).

Intestinal integrity was assessed using the lactulose:mannitol (L:M) intestinal permeability test. The test dose consisted of lactulose (4 g; Sandoz, Camberley, Surrey, UK) and mannitol (1 g; Sigma–Aldrich, Gillingham, Kent, UK) made up to 20 ml with water. Following the completion of the 2-h urine collection period, the subjects were instructed to drink the test solution and their urine was collected during the following 5 h into containers containing a few drops of chlorhexidine antiseptic solution (5% w/v, Holden Medical, Lelystad, The Netherlands) to prevent bacterial contamination and sugar degradation. At the end of the 5-h urine collection period, urine aliquots were frozen at −20 °C, transported to MRC HNR on dry ice, stored at −20 °C and assayed using in-house methods described elsewhere (20, 28). A mannitol absorption of <14% was taken to indicate malabsorption and partial villus atrophy, and a lactulose absorption >1% was taken to indicate disaccharide hyperpermeability (29).

### 25OHD half-life

25OHD-*t*_1/2_ was determined using the 25OHD terminal slope principle described elsewhere (30) in a subset of children (BD *n*=12, LC+*n*=12, and LC− *n*=13). 3-^2^H-25-hydroxyvitamin D_3_ (6, 19, 19-d_3_) (d_3_-25OHD_3_) (Sigma–Aldrich; 97 atom % D, 98% (CP)) was dissolved in vegetable oil and a dose (40 nmol) was given on a small piece of bread after the fasting blood sample was collected (day 1). Further fasting LiHep-blood samples were collected on days 6, 9, 21, 24 and 30. Plasma samples were frozen at −70 °C and analysed at MRC HNR. The samples were subjected to solid-phase extraction and derivatisation with 50 μl 4-phenyl-1,2,4-triazoline-3,5-dione (0.5 mg/ml) solution in acetonitrile as described elsewhere (30). Analyses were carried out using a UPLC system (LC-20 AS, Shimadzu Ltd., Milton Keynes, UK) interfaced to an ABSciex 5500 QTRAP mass spectrometer (MDS Analytical Technologies, Concord, ON, Canada). The MS was operated in electrospray ionisation (ESI) positive mode with multiple reaction monitoring (MRM). Derivatised sample extracts (10 μl) were injected into an Acquity UPLC BEH C_18_ column (2.1×100 mm, 1.7 μm, Waters, Elstree, Hertfordshire, UK) maintained at 45 °C. Mobile phases were eluent A (0.1% formic acid and 5 mM methylamine in H_2_O) and eluent B (0.1% formic acid in a mixture of methanol:H_2_O:acetonitrile (97:2:1, v/v/v)). The software used for system control and chromatogram integration was Analyst version 1.5.2 (Applied Biosystems, Concord, ON, Canada). 25OHD-*t*_1/2_ was calculated from the terminal slope of the disappearance of d_3_-25OHD_3_ using the following equation *t*_½_=ln(2)/k_B_, where k_B_ is the natural logarithm of the slope of the line of best fit calculated from d6 to d30 (30).

### Statistical analyses and calculations

All the statistical analyses were carried out using DataDesk 6.3 (Data Description, Inc., Ithaca, NY, USA). Positively skewed variables were transformed using natural logarithms before statistical tests. Summary statistics are expressed as mean (s.d.) for normally distributed variables or as geometric mean (−1s.d., +1s.d.) for skewed variables. For continuous variables, group differences were assessed using age- and sex-corrected regression analysis for two groups or with ANOVA followed by two-sample Student's *t*-tests. For categorical variables, group differences were identified using *χ*^2^ tests. For analysis of predictors of 25OHD-*t*_1/2_, I-FGF23 and C-FGF23, multiple regression with stepwise backwards elimination was conducted without correcting for age or sex. With the variables expressed as natural logarithms, the coefficient (s.e.m.) for a group difference ×100 closely equates to the difference expressed as a sympercent (s.e.m.) (31). Longitudinal analysis of C-FGF23 was conducted using conditional regression, whereby the change (Δ=ln Previous_C-FGF23_−ln Baseline_C-FGF23_) was the dependent variable and baseline C-FGF23 concentration and baseline age were corrected for. In this exploratory study, *P*≤0.05 was considered to be statistically significant and no adjustments were made to account for the number of tests carried out.

Glomerular filtration rate (eGFR) was estimated in the following way using plasma cys C (32):


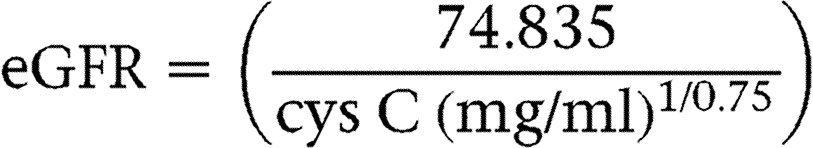


Tubular maximal reabsorption of P (TmP:GFR) was calculated in the following way (33):


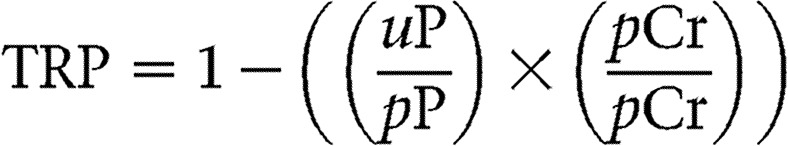


for TRP ≤0.86, then TmP:GFR=TRP×*p*P, or for TRP >0.86, then:


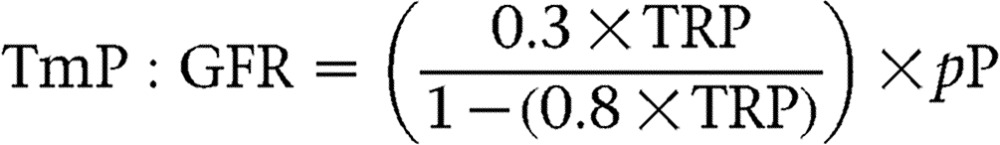


The percentage absorption of L was calculated in the following way:


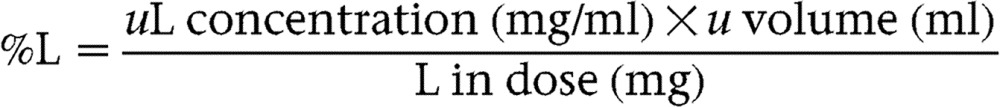


%M was calculated in the same way.

## Results

### Anthropometry

BD children no longer had visible signs of bone deformities, and there was no significant difference in age, height, sitting height or weight between the three groups (Table 1). BD children had a greater percentage of total body fat and percentage of trunk fat mass than LC children (%total body fat: BD=16.9 (4.5), LC+=15.6 (3.1), and LC−=14.7 (3.7); ANOVA *P*=0.01; %trunk fat mass: BD=12.4 (3.5), LC+=9.9 (2.3), and LC−=9.7 (2.9); ANOVA *P*=0.004) (Table 1).

### Concentration and predictors of FGF23

**Figure 1:**
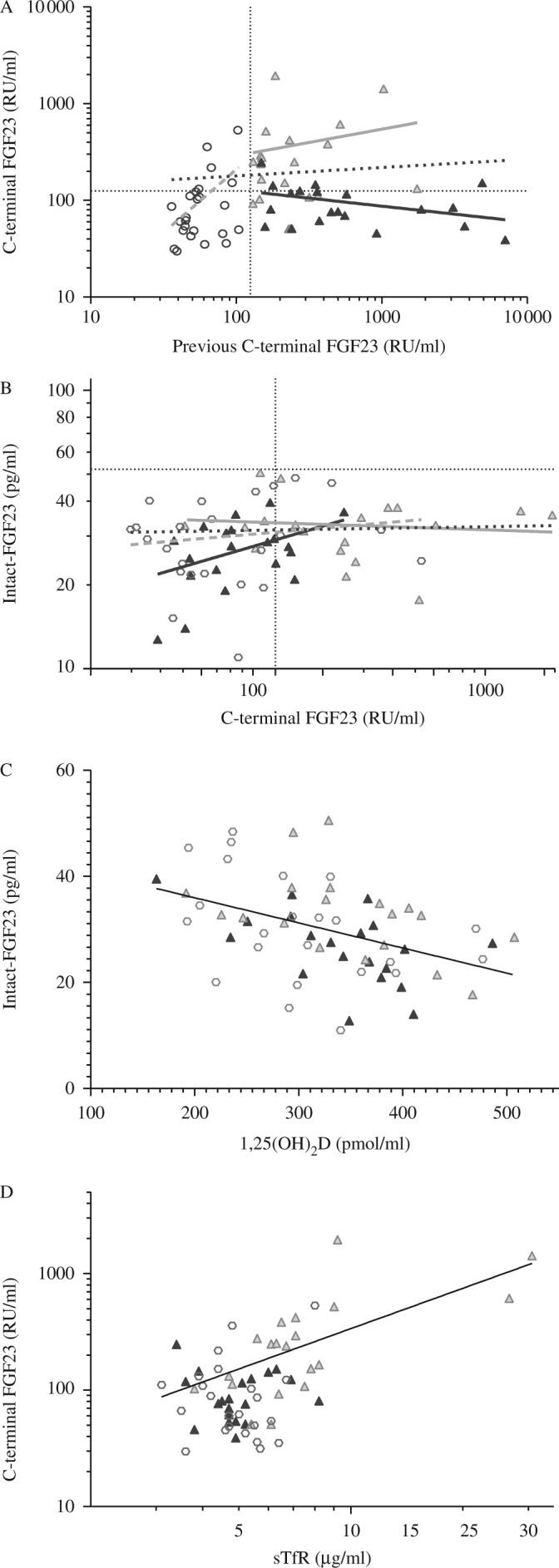
Scatterplot of C-terminal FGF23 (C-FGF23) concentrations at baseline and follow-up (A); C-FGF23 and intact FGF23 concentrations at follow-up (B);1,25(OH)_2_D and I-FGF23 concentrations (C); and sTfR and C-FGF23 (D) concentrations by group. BD black triangle, history of rickets and a previously measured elevated C-FGF23 concentration; LC+ grey triangle, local controls with a previously measured high C-FGF23 concentration; LC−, local controls with a previously measured normal C-FGF23 concentration. Dotted lines at 125 RU/ml and 52 pg/ml are upper levels of normal for C-terminal and intact-FGF23 concentrations respectively. Equations of the line: 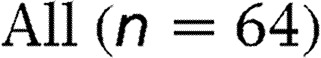 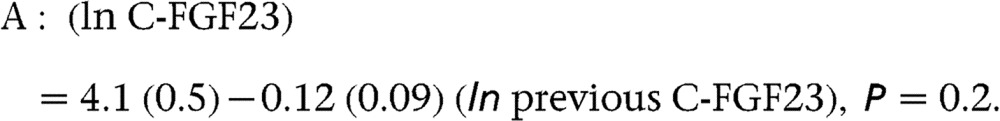 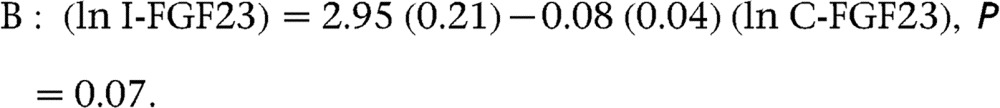 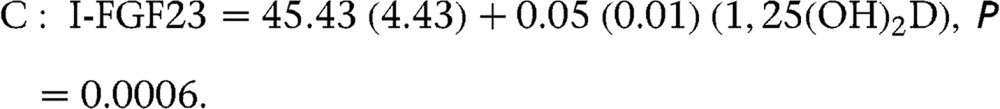 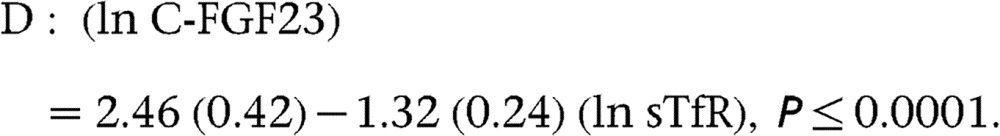 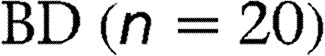 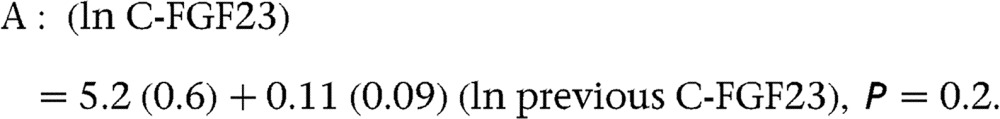 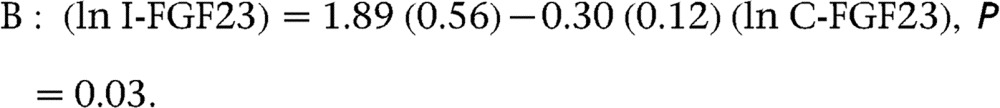 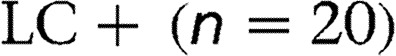 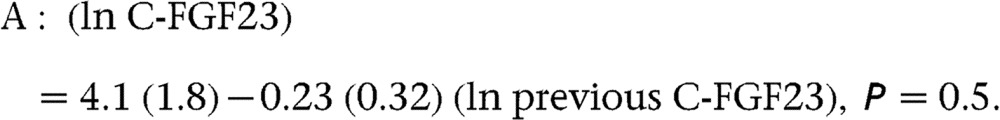 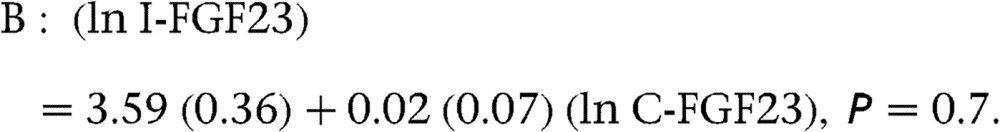 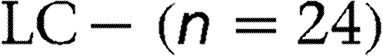 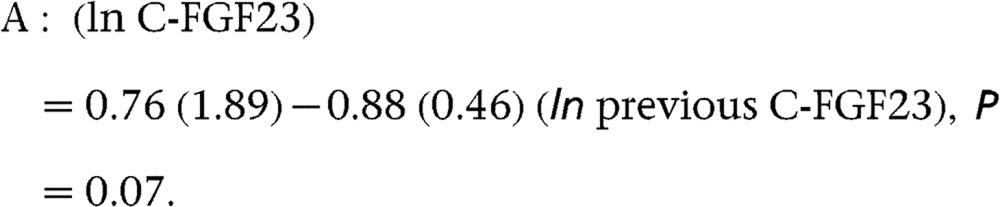 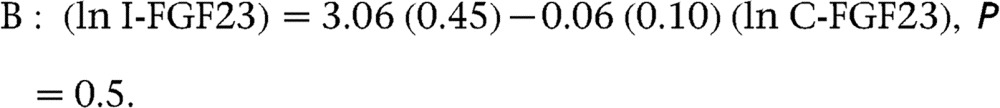

In BD children, C-FGF23 concentration had decreased significantly since the previous C-FGF23 concentration measurement (obtained mean (s.d.) 4.9 (0.5) years before). The percentage of BD children with an elevated C-FGF23 concentration (>125 RU/ml) decreased from 100 to 25% (BD geometric mean (−1s.d., +1s.d.) C-FGF23: from 536 (167, 1715) to 85 (53, 140) RU/ml; conditional regression *P*=0.01) (Fig. 1A). By contrast, there was no significant change in C-FGF23 concentration in LC+ children (from 252 (125, 509) to 230 (87, 608) RU/ml; conditional regression *P*=0.2). C-FGF23 concentration remained elevated in 70% of the LC+ children. There was no significant change over time in LC− children (58 (42, 79) to 79 (37, 170) RU/ml; conditional regression *P*=0.3). Consequently, C-FGF23 concentration at this follow-up was significantly higher in LC+ children than in either BD or LC− children (ANOVA *P*≤0.0001), but was not significantly different between BD and LC− children (Table 1).

All children had an I-FGF23 concentration within the normal range, but I-FGF23 concentration was higher in LC+ children than in BD children (BD=25.7 (19.2, 34.5), LC+=34.3 (22.8, 51.5), and LC−=28.2 (19.6, 40.7) pg/ml; *P*=0.05) (Table 1).

C-FGF23 concentration did not significantly correlate with I-FGF23 concentration when all the groups were combined (*n*=64) (coefficient % (s.e.m.): 8.2 (4.0) %; *R*^2^=5.3%; *P*=0.07). However, C-FGF23 was a significant predictor of I-FGF23 concentration in BD children (coefficient % (s.e.m.): 30 (12) %; *R*^2^=24%; *P*=0.03) but not in LC children (coefficient % (s.e.m.): LC+=−2.5 (6.5)%; *R*^2^=0.8%; *P*=0.7; LC−=6.4 (10.3)%; *R*^2^=1.7%; *P*=0.5) (Fig. 1B).

There was biochemical evidence that LC+ children had poorer iron status compared with the other two groups as demonstrated by higher ZnPP and sTfR concentrations compared with those in BD and LC− children (Table 1).

1,25(OH)_2_D was the strongest predictor of I-FGF23 concentration (*n*=64; coefficient % (s.e.m.): −4.7 (1.0)%; *R*^2^=18.1%; *P*=0.0006) (Fig. 1C) but not of C-FGF23 concentration (*n*=64; coefficient % (s.e.m.):−25.4 (44.2)%; *R*^2^=0.5%; *P*=0.6) in univariate analyses. sTfR was the strongest predictor of C-FGF23 concentration (*n*=64; coefficient % (s.e.m.): 132 (24) %; *R*^2^=33.2%; *P*≤0.0001) (Fig. 1D), but it did not predict I-FGF23 concentration (*n*=64; coefficient % (s.e.m.): 8.6 (10.5) %; *R*^2^=1.1%; *P*=0.4) in univariate analyses.

Using stepwise elimination in multivariate analysis, 1,25(OH)_2_D and 25OHD were found to be the only predictors of I-FGF23 concentration (among C-FGF23, PTH, P, *i*Ca, sTfR, Ferr, Hb, ZnPP, TmP:GFR, eGFR, and dietary Ca and phosphorus intake, which were not significant). 1,25(OH)_2_D and 25OHD remained in the model and together predicted 28.5% of the variation in I-FGF23 concentration, whereas only sTfR and Ferr remained in the model for C-FGF23 and together predicted 45.0% of the variation in C-FGF23 concentration.

### Dietary Ca intake and factors affecting Ca absorption

Mean dietary Ca intake was 265 (12.6) mg/day and did not differ significantly between the groups (mean (s.d.) mg/day: BD=259 (129), LC+=273 (133), and LC−=263 (108); ANOVA *P*=0.9). There were also no significant differences in phosphorus (Table 1), energy or protein intake (data not shown).

Eighty-five percentage of all children were identified as having an *H. pylori* infection, and there was no difference in the prevalence of infection between the groups (*H. pylori* infection %: BD=84%, LC+=80%, and LC−=91%; *χ*^2^ *P*=0.6). L:M test indicated signs of intestinal malabsorption in the majority of children (%M <14 in 88% of the children), and this distribution was similar across all the three groups (%M ≤14: BD=90%, LC+=84%, and LC−=92%; *χ*^2^ *P*=0.7). The percentage recovery of L was within the normal range (<1%) in the majority of children (%L <1 in 96% of the children), and this distribution was similar across all the three groups (%L <1: BD=100%, LC+=89%, and LC−=100%; *χ*^2^*P*=0.1).

### 25OHD half-life

There were no significant differences in anthropometry, biochemical profile or dietary intake between those who did and did not participate in the 25OHD-*t*_1/2_ analysis, with the exception of plasma alb and AGP concentrations (alb concentration was ∼1.6 g/l higher and AGP concentration ∼2.2 μmol/l lower in those who did participate in the 25OHD-*t*_1/2_ analysis compared with those in children who did not).

25OHD-*t*_1/2_ was significantly shorter in BD children (*n*=12) than in LC− children (*n*=13) (24.5 (6.1) and 31.5 (11.5) days respectively; *P*=0.05). 25OHD-*t*_1/2_ was not significantly different between LC+ (*n*=12) and BD children (27.8 (6.4) and 24.5 (6.1) days respectively; *P*=0.3) or LC+ and LC− children (27.8 (6.4) and 31.5 (11.5) days respectively; *P*=0.3). No significant predictors of 25OHD-*t*_1/2_ were identified from among plasma C-FGF23, I-FGF23, *i*Ca, P, PTH, 1,25(OH)_2_D, 25OHD or dietary Ca intake in multiple regression analysis.

## Discussion

Elevated plasma C-FGF23 concentrations have been recorded in Gambian children with putative Ca-deficiency rickets (1) and, at a lower prevalence, in apparently healthy children from the local community (2). This longitudinal study of children previously identified to have an elevated C-FGF23 concentration, with and without putative Ca-deficiency rickets (1), revealed that C-FGF23 concentration, as well as other biochemical abnormalities, had normalised in the children with resolved rickets (BD), whereas C-FGF23 concentration remained unchanged in apparently healthy controls. Local controls with a previously measured elevated C-FGF23 concentration (LC+) had an elevated concentration at follow-up, whereas local controls with a previously measured normal C-FGF23 concentration (LC−) had a normal concentration.

I-FGF23 concentration was higher in LC+ children than in BD children, but was within the normal ranges in all the children. Interestingly, C-FGF23 and I-FGF23 concentrations correlated positively in the BD group, but did not correlate in either of the LC groups or when the three groups were combined. As well as a persistently elevated C-FGF23 concentration, LC+ children also had poorer iron status compared with the other children. The strong association between iron status and C-FGF23 concentration in the LC+ group may suggest a role of iron in FGF23 metabolism.

One of the main findings of this study is that 25OHD-*t*_1/2_ in children with a history of rickets (BD) was shorter than that in children from the local community (LC−). Despite the facts that their biochemical and bone abnormalities had normalised and that the children no longer had significantly elevated PTH, 1,25(OH)_2_D, C-FGF23 or TALP concentrations or visible signs of bone deformities, 25OHD-*t*_1/2_ in children with a history of rickets was ∼7 days shorter than that in the local community children, suggestive of a long-standing increased expenditure of 25OHD. It is possible that BD children had increased activities of CYP27B1 and/or CYP24A1, which would in turn increase the rate of production of 1,25(OH)_2_D and 24,25(OH)_2_D and 1,24,25(OH)_3_D respectively. However, we cannot test this hypothesis in the present study because there are insufficient samples remaining and because we do not yet have an analytical method that is able to determine the concentration of downstream metabolites of 25OHD. Despite differences in 25OHD-*t*_1/2_, there was no difference in 25OHD concentrations between BD and LC− children. This might be explained by the likelihood that endogenous vitamin D production was not limiting in these children who were living in a tropical country with year-round abundant UVB-containing sunshine.

Another finding of this study is that in addition to the small amounts of Ca present in the children's diet, there was a high prevalence of factors that decrease Ca absorption, such as infection and enteropathy, which may further reduce Ca supply. This study demonstrated that over 80% of the children were infected with *H. pylori*. This is in keeping with previous Gambian studies that have shown that the prevalence of *H. pylori* infection increases to ∼80% throughout infancy (<2.5 years) (34). Eighty-eight percentage of the children showed signs of intestinal malabsorption as indicated by a low percentage recovery of mannitol (a passively absorbed sugar). Intestinal malabsorption is often observed in populations in which diarrhoeal diseases are common. Numerous diarrhoeal episodes are thought to result in partial villus atrophy, which leads to a reduction in intestinal absorption ability of minerals such as Ca.

I-FGF23 and C-FGF23 concentrations had different biochemical predictors and were poorly correlated with each other. 1,25(OH)_2_D was the strongest negative predictor of I-FGF23 concentration, in line with the known suppressive action of FGF23 on CYP27B1. By contrast, sTfR, high concentrations of which describe poor iron status, and not 1,25(OH)_2_D was the strongest predictor of C-FGF23 concentration. This supports previous reports from the Gambia and elsewhere showing an association between C-FGF23 concentration and various markers of poor iron status (2, 14, 35) and suggests that the interpretation of data from C-FGF23 assays should be made with caution in populations with a high prevalence of iron deficiency. A recent study in rats has shown that iron deficiency had effects on *Fgf23* gene expression similar to those of oxygen deprivation, suggesting a mechanism that iron deficiency may result in hypoxia resulting in an increased *Fgf23* gene expression due to hypoxia-induced factors (15). It has been suggested that an increased production of FGF23 caused by poor iron status is balanced by an increased cleavage of the I-FGF23 protein (16, 17). This would result in increased concentrations of cleaved and biologically inactive C- and N-terminal fragments but an unchanged concentration of the I-FGF23 and active FGF23 hormone. Such a mechanism would explain why the LC+ children showed no signs of phosphate wasting or rickets, despite having prolonged elevated concentrations of C-FGF23. An additional element to consider is the possible antagonistic effects of C-FGF23 fragments on the FGFR. Goetz *et al*. (10) showed in otherwise healthy mice that an injection of a recombinant C-terminal fragment results in hyperphosphataemia due to a decreased urinary P excretion. However, the question remains as to why LC+ children have a higher prevalence of iron deficiency than their peers. This may be an indication of a genetic disorder of iron metabolism such as thalassemia or sickle cell trait, both of which are known to be prevalent in Gambia (36). The initial cause of an elevated C-FGF23 concentration in BD children and its role in the pathogenesis of rickets are unclear. Moreover, differences in FGF23 concentrations do not seem to affect 25OHD-*t*_1/2_, despite the described roles of FGF23 in CYP enzyme activity (6, 7).

In summary, this study demonstrated that elevated plasma C-FGF23 concentrations normalised over time in Gambian children with a history of rickets but not in apparently healthy local children, suggesting that the aetiology of a raised FGF23 concentration is different in these two groups. This study adds to the growing literature that C-FGF23 and I-FGF23 assays are not always comparable (37) and reflect different components of the FGF23 pathway and that markers of iron status are the strongest predictors of C-FGF23 concentration but not of I-FGF23 concentration. Finally, this study demonstrated that children with resolved rickets had a shorter 25OHD-*t*_1/2_ than local children, suggesting a long-standing increased expenditure of 25OHD.

## Figures and Tables

**Table 1:** Differences in anthropometry, biochemical profile and dietary indices by group.

**Dependent**	**BD**	**LC+**	**LC−**	**ANOVA**
Variable	*n*=20	*n*=20	*n*=24	*P* value
Anthropometry				
Age (years)	11.9 (2.4)	12.3 (2.5)	12.3 (2.3)	0.8
Sex (F/M)	7/13	12/8	7/17	0.1
Height (cm)	141.4 (14.9)	142.7 (14.3)	143.7 (12.7)	0.8
Sitting height (cm)	70.8 (6.2)	71.4 (5.4)	72.2 (6.0)	0.6
Weight (kg)	32.2 (9.7)	31.5 (9.5)	32.3 (9.2)	0.4
BMI (kg/m^2^)	15.7 (2.0)^b^	15.0 (1.9)^a^	15.3 (2.1)	0.1
% Fat Tanita	16.9 (4.5)^b^	15.6 (3.1)^a^	14.7 (3.7)	0.01
% Trunk fat mass	12.4 (3.5)^b,c^	9.9 (2.3)^a^	9.7 (2.9)^a^	0.004
Plasma biochemistry				
iCa (7.4) (mmol/l)	1.13 (0.02)	1.14 (0.03)	1.13 (0.03)	0.7
Ca (mmol/l)	2.32 (0.04)	2.34 (0.07)	2.32 (0.05)	0.2
25OHD (nmol/l)	61.1 (16.8)	62.1 (16.5)	57.8 (13.8)	0.6
25OHD-*t*_1/2_ (days)	24.5 (6.1)^c^	27.8 (6.4)	31.5 (11.5)^a^	0.09
1,25(OH)_2_D (pmol/l)	339.9 (71.4)	352.7 (84.0)	301.5 (79.4)	0.1
PTH (pg/ml)	41.0 (19.4)	39.9 (18.8)	35.9 (21.9)	0.6
PO_4_^3−^ (mmol/l)	1.48 (0.17)	1.47 (0.17)	1.56 (0.15)	0.3
C-FGF23*(RU/ml)	84.7 (53.7, 140.0)^b^	229.8 (86.9, 607.4)^a,c^	78.9 (37.3, 166.9)^b^	0.0001
I-FGF23*(pg/ml)	25.7 (19.2, 34.5)^b^	34.3 (22.8, 51.5)^a^	28.2 (19.6, 40.7)	0.05
TALP (U/l)	321.6 (73.2)	348.9 (108.7)	362.3 (107.2)	0.4
Cys C (mg/l)	0.81 (0.15)^c^	0.86 (0.15)^c^	0.99 (0.17)^a,b^	0.002
Cr (μmol/l)	47.2 (8.5)	43.3 (7.0)	46.5 (10.4)	0.2
Albumin (g/l)	38.5 (2.3)	38.6 (2.9)	37.4 (2.4)	0.2
Hb*(g/dl)	13.4 (12.4, 14.4)	12.6 (10.4, 15.3)	13.3 (12.3, 14.3)	0.2
ZnPP*(μmol/mol per haem)	69.4 (51.2, 93.9)^b^	99.8 (66.2, 150.7)^a,c^	68.8 (50.2, 94.2)^b^	0.008
Ferr*(μg/l)	31.6 (15.5, 40.7)	20.2 (8.30, 48.9)	29.7 (17.0, 51.9)	0.2
sTfR*(μg/ml)	4.91 (3.96, 6.09)^b^	7.45 (4.48, 6.09)^a,c^	4.89 (3.90, 6.11)^b^	0.0001
AGP (μmol/l)	17.0 (5.6)	18.3 (5.7)	18.2 (3.9)	0.5
CRP*(mg/dl)	2.12 (0.74, 6.07)	2.32 (0.99, 4.45)	2.38 (0.99, 5.48)	0.9
Mg (mmol/l)	0.85 (0.06)	0.85 (0.07)	0.84 (0.08)	0.5
eGFR and mineral excretion				
eGFR (ml/min)	102.2 (22.6)^c^	95.5 (22.8)	78.8 (17.2)^a^	0.002
TmP:GFR (mmol/l)	1.82 (0.29)^c^	1.88 (0.20)	1.98 (0.21)^a^	0.08
2-h *u*PO_4_^3−^:Cr*(mol/mol)	1.29 (0.80, 2.08)	0.93 (0.47, 1.83)	1.20 (0.87, 1.64)	0.1
2-h *u*Ca:Cr*(mol/mol)	0.35 (0.15, 0.81)	0.29 (0.16, 0.51)	0.87 (0.46, 1.65)	0.4
24-h *u*PO_4_^3^:Cr*(mol/mol)	1.98 (1.44, 2.74)	1.92 (1.44, 2.55)	1.83 (1.18, 2.83)	0.7
24-h *u*Ca:Cr*(mol/mol)	0.32 (0.16, 0.67)	0.24 (0.14, 0.42)	0.26 (0.13, 0.50)	0.5
Two-day weighed dietary intake				
Ca (mg)	259 (129)	273 (133)	263 (108)	0.9
Phosphorus (mg)	679 (267)	722 (315)	671 (260)	0.5
Ca/P (mol/mol)	0.30 (0.11)	0.30 (0.12)	0.32 (0.10)	0.8

BD, history of rickets and a previously measured elevated C-terminal FGF23 (C-FGF23) concentration; LC+, local controls with a previously measured high C-FGF23 concentration; and LC−, local controls with a previously measured normal C-FGF23 concentration. All measurements were conducted on the entire dataset (*n*=64), with the exception of 25OHD half-life (25OHD-*t*_1/2_), which was conducted on a subset (BD: *n*=12, LC+: *n*=12, and LC−: (*n*=13)). For normally distributed data, the results are mean (s.d.); for positively skewed data (denoted by *), the results are geometric mean (−1s.d., +s.d.). The ANOVA *P* value is age- and sex-adjusted with the exception of the sex variable, which is a *χ*^2^ *P* value. Significant differences between the groups as determined by two-sample Student's *t*-tests are denoted by a superscript, i.e. ^a^= significantly different from BD, ^b^=significantly different from LC+, and ^c^=significantly different from LC−.
